# Artificial Neural Networks to Solve the Singular Model with Neumann–Robin, Dirichlet and Neumann Boundary Conditions

**DOI:** 10.3390/s21196498

**Published:** 2021-09-29

**Authors:** Kashif Nisar, Zulqurnain Sabir, Muhammad Asif Zahoor Raja, Ag Asri Ag Ibrahim, Joel J. P. C. Rodrigues, Samy Refahy Mahmoud, Bhawani Shankar Chowdhry, Manoj Gupta

**Affiliations:** 1 Faculty of Computing and Informatics, Universiti Malaysia Sabah, Jalan UMS, Kota Kinabalu Sabah 88400, Malaysia; awgasri@ums.edu.my; 2 Department of Mathematics and Statistics, Hazara University, Mansehra 21120, Pakistan; zulqurnain_maths@hu.edu.pk; 3 Future Technology Research Center, National Yunlin University of Science and Technology, 123 University Road, Section 3, Douliou, Yunlin 64002, Taiwan; rajamaz@yuntech.edu.tw; 4 PPGEE, Federal University of Piauí (UFPI), Teresina 64049-550, Brazil; joeljr@ieee.org; 5 Covilhã Delegation, Instituto de Telecomunicações, 6201-001 Covilhã, Portugal; 6 GRC Department, Faculty of Applied Studies, Jeddah, King Abdulaziz University, Jeddah 21589, Saudi Arabia; srhassan@kau.edu.sa; 7 NCRA Condition Monitoring Systems Lab, Mehran University of Engineering and Technology, Jamshoro 76020, Pakistan; bhawani.chowdhry@faculty.muet.edu.pk; 8 Department of Electronics and Communication Engineering, JECRC University Jaipur, Rajasthan 303905, India; manojgupta35@yahoo.co.in

**Keywords:** singular, Neumann–Robin, Dirichlet, sequential quadratic, genetic algorithm, neuron analysis

## Abstract

The aim of this work is to solve the case study singular model involving the Neumann–Robin, Dirichlet, and Neumann boundary conditions using a novel computing framework that is based on the artificial neural network (ANN), global search genetic algorithm (GA), and local search sequential quadratic programming method (SQPM), i.e., ANN-GA-SQPM. The inspiration to present this numerical framework comes through the objective of introducing a reliable structure that associates the operative ANNs features using the optimization procedures of soft computing to deal with such stimulating systems. Four different problems that are based on the singular equations involving Neumann–Robin, Dirichlet, and Neumann boundary conditions have been occupied to scrutinize the robustness, stability, and proficiency of the designed ANN-GA-SQPM. The proposed results through ANN-GA-SQPM have been compared with the exact results to check the efficiency of the scheme through the statistical performances for taking fifty independent trials. Moreover, the study of the neuron analysis based on three and 15 neurons is also performed to check the authenticity of the proposed ANN-GA-SQPM.

## 1. Introduction

The singular nonlinear models that are governed with the Emden–Fowler types of equations are considered to be a hot topic for the researchers due to their massive applications in population evolution, relativistic or fluid mechanics, chemical reactor systems, and pattern structure [1,2,3,4,5]. The EF is one of the popular models due to the singular point its the origin. Some well-known applications of the singular models are the electromagnetic structure, oscillating magnetic fields, stellar structure, catalytic diffusion reactions, continuous isotropic media, isothermal gas spheres, and classical mechanics [6,7,8,9,10,11,12]. The typical form of the EF model, which is basically a second order singular differential equation, is given as [13,14,15,16]:

(1) $$\frac{d^{2}z}{d\Omega^{2}}+\frac{\chi }{\Omega }\frac{dz}{d\Omega }+h(\Omega )g(z)=u(\Omega ),\;z(0)=a,\;\frac{dz(0)}{d\Omega }=0.$$

The model (1) takes the form of Lane–Emden equation for h(Ω)= 1, given as:

(2) $$\frac{d^{2}z}{d\Omega^{2}}+\frac{\chi }{\Omega }\frac{dz}{d\Omega }+g(z)=u(\Omega ),\;z(0)=a,\;\frac{dz(0)}{d\Omega }=0.$$

where χ represents the shape factor, u(Ω) is the forcing function, h(Ω) and g(z) are the functions of Ω and *z*, respectively. The purpose of this research work is to solve the singular equations involving Neumann–Robin, Dirichlet, and Neumann boundary conditions using a novel computing framework that is based on the artificial neural network (ANN), global search genetic algorithm (GA), and local search sequential quadratic programming method (SQPM), i.e., ANN-GA-SQPM. The general form of the singular models, along with the Neumann–Robin, Dirichlet, and Neumann boundary conditions, is written as [17]:

(3) $$\frac{d^{2}z}{d\Omega^{2}}+\frac{\chi }{\Omega }\frac{dz}{d\Omega }+g(z)=u(\Omega ),\;\begin{array}{l} z(0)=A_{1},\;\;\;\;\;z(1)=B_{1},\;\\ \frac{dz(0)}{d\Omega }=A_{2},\;\;\;\;\;\;\frac{dz(1)}{d\Omega }=B_{2},\;\\ \frac{dz(0)}{d\Omega }=0,\;\;\;\;\;A_{3}z(1)+B_{3}\frac{dz(1)}{d\Omega }=C_{1} \end{array}$$

where $A_{1},B_{1},\;A_{2},B_{2},A_{3},\;B_{3}$, and $C_{1}$ are the real constants. For the numerical outcomes of the singular system, a variety of applications have been investigated in the references [18,19,20]. All of these stated approaches have their separate perks and importance, as well as confines and disadvantages. Alongside these conventional schemes, the stochastic design of the computational numerical heuristic or swarming techniques appears to be capable and proficient in integrating the singular systems by operating the universal approximation capability of ANNs along with local/global techniques. The nonlinear prey-predator system [21], nonlinear singular Thomas–Fermi system [22], SITR nonlinear based COVID model [23,24], periodic singular differential system [25,26], dengue fever model [27], multi-singular systems [28], HIV infection model [29], nonlinear singular functional differential model [30,31], heat conduction system in human head [32], and mosquito dispersal model [33] are some recent submissions of the stochastic solvers. When considering these influences, the authors are inspired to present the solution of the singular models involving the Neumann–Robin, Dirichlet, and Neumann boundary conditions using the ANN-GA-SQPM. Some novel characteristics of the ANN-GA-SQPM are briefly provided as:

- A pioneering framework using the integrated computational ANN-GA-SQPM is provided to solve the singular model involving the Neumann–Robin, Dirichlet, and Neumann boundary conditions.
- The performance of the computational ANN-GA-SQPM is observed using a small and large number of neurons.
- The matching of the results that were obtained by the proposed computational ANN-GA-SQPM with the exact solutions authenticate the value in terms of convergence and precision.
- The absolute error (AE) is found in good measure for each problem of the singular model.
- The verification of the ANN-GA-SQPM is authorized from the statistical exploration on multiple executions for 10 neurons based on the performance of Variance Account For (VAF), Nash Sutcliffe Efficiency (NSE), and Theil’s Inequality Coefficient (TIC).
- Besides the equitable precise solutions of the system, the easy understanding, smooth operations, robustness, and comprehensive stability are other valued merits.

## 2. Methodology: ANN-GA-SQPM

The design structure is presented in two phases to solve the singular model. An error function is introduced and the hybridization procedures of GA-SQPM is provided.

### 2.1. ANNs Modeling

In order to solve the singular models involving the Neumann–Robin, Dirichlet, and Neumann boundary conditions that accumulated with feed-forward ANNs, $\hat{z}(\Omega )$ is the continuous mapping form of the solution together with the log-sigmoid function $Q(\Omega )=1/\left(1+\exp(-\Omega )\right)$ given as:

(4) $$\begin{array}{l} \hat{z}(\Omega )=\sum_{i=1}^{k}r_{i}Q(w_{i}\chi +s_{i})=\sum_{i=1}^{k}r_{i}{\left(1+e^{-(w_{i}\chi +s_{i})}\right)}^{-1},\\ \frac{d\hat{z}}{d\chi }=\sum_{i=1}^{k}r_{i}\frac{d}{d\chi }Q(w_{i}\chi +s_{i})=\sum_{i=1}^{k}r_{i}w_{i}e^{-(w_{i}\chi +s_{i})}{\left(1+e^{-(w_{i}\chi +s_{i})}\right)}^{-2},\\ \frac{d^{2}\hat{z}}{d\chi^{2}}=\sum_{i=1}^{k}r_{i}\frac{d^{2}}{d\chi^{2}}Q(w_{i}\chi +s_{i})=\sum_{i=1}^{k}r_{i}w_{i}^{2}\left(\frac{2e^{-2(w_{i}\chi +s_{i})}}{{\left(1+e^{-(w_{i}\chi +s_{i})}\right)}^{3}}-\frac{e^{-(w_{i}\chi +s_{i})}}{{\left(1+e^{-(w_{i}\chi +s_{i})}\right)}^{2}}\right), \end{array}$$

where $r=[r_{1},r_{2},r_{3},\ldots r_{k}],\;\;w=[w_{1},w_{2},w_{3},\ldots ,w_{k}]$, and $s=[s_{1},s_{2},s_{3},\ldots ,s_{k}]$ are the unknown weights. An error function for solving the singular model (3) is given as:

(5) $$\xi_{Fit}=\xi_{Fit-1}+\xi_{Fit-2},$$

where the construction of the error functions $\xi_{Fit-1}$ and $\xi_{Fit-2}$ is on the basis of the singular model and the Neumann–Robin, Dirichlet, and Neumann boundary conditions, respectively, given as:

(6) $$\xi_{Fit-1}=\frac{1}{N}\sum_{k=1}^{N}\left(\frac{d^{2}{\hat{z}}_{k}}{d\Omega_{k}^{2}}+\frac{\chi }{\Omega_{k}}\frac{d{\hat{z}}_{k}}{d\Omega_{k}}+g({\hat{z}}_{k})-u_{k}\right),\;\;\;0\leq \Omega_{k}\leq 1,$$

(7) $$\xi_{Fit-2}=\frac{1}{2}{\left({\hat{z}}_{0}-A_{1}\right)}^{2}+\frac{1}{2}{\left({\hat{z}}_{N}-B_{1}\right)}^{2},$$

(8) $$\xi_{Fit-3}=\frac{1}{2}{\left({{\hat{z}}^{\prime }}_{0}-A_{2}\right)}^{2}+\frac{1}{2}{\left({{\hat{z}}^{\prime }}_{N}-B_{2}\right)}^{2},$$

(9) $$\xi_{Fit-4}=\frac{1}{2}{\left({{\hat{z}}^{\prime }}_{0}\right)}^{2}+\frac{1}{2}{\left(A_{3}{\hat{z}}_{N}-B_{3}{{\hat{z}}^{\prime }}_{N}-C_{1}\right)}^{2},$$

where $Nh=1,\;\;{\hat{z}}_{k}=z(\chi_{k}),\;\;\;g({\hat{z}}_{k})=g(\chi_{k}),\;\;\;\;u_{k}=u(\chi_{k})\;\;\mathrm{and}\;\;\;z_{k}=kh.$

### 2.2. Optimization Process: GA-SQPM

The optimization performance of the ANNs is accomplished through the hybridization procedures of GA-SQPM.

GAs are applied to solve both constrained/unconstrained optimization models based on the natural selection process. GAs are implemented frequently to transform the population of individual solutions that solve an assortment of systems using the optimization procedures, where the fundamental optimization schemes fail, e.g., non-differentiable systems, stochastic models, and highly nonlinear systems. GA is applied in various fields of technologies, applied sciences, and engineering that work through its reproduction operators. In recent years, GA and PSO based heuristic optimization solvers have been applied in transportation planning and logistics management [34], microgrid energy management systems [35], optimization of multimodal functions [36], mobile position estimation problem [37], the vehicle routing problem in cloud implementation [38], satellite formation reconfiguration [39], and the optimization of multi-objective energy models [40].

SQPM is considered to be very important in the process of optimization. SQPM is known as a local search scheme and it has been implemented to solve constrained/unconstrained models. In recent years, SQPM has been implemented in the sizing and location of DGs [41], optimal gait based on bipedal robots through nonlinear system of predictive control [42], optimal organization of directional overcurrent communication incorporating spread generation [43], second order prediction differential system [44], central air-conditioning optimization [45], and flight vehicle management [46].

One can use the process of hybridization with the local search scheme to help overcome the sluggishness and laziness associated with controlling the global scheme. Table 1 presents the pseudocode of the GA-SQPM.

## 3. Results and Discussion

The current section provides details of the nonlinear singular differential model based on the Neumann–Robin, Dirichlet, and Neumann boundary conditions using the designed framework of ANN-GA-SQPM. Two problems based on nonlinear singular systems with the Dirichlet boundary condition while one problem each for Neumann–Robin and Neumann boundary conditions, respectively, are implemented in order to evaluate the performance of the designed ANN-GA-SQPM. The details of the results comparison, AE, performance measures, and weight plots, along with statistical observations, are also presented.

**Problem 1:** Consider the following singular Lane–Emden nonlinear model along with Dirichlet boundary conditions, which are written as:

(10) $$\begin{array}{l} \frac{d^{2}z}{d\Omega^{2}}+\frac{0.5}{\Omega }\frac{dz}{d\Omega }+e^{2z}=0.5e^{z},\\ z(0)=\log(2),\;\;z(1)=0. \end{array}$$

The exact solution for the above equation is $\ln\left(\frac{2}{1+\Omega^{2}}\right)$. The error function is given as:

(11) $$\xi_{Fit}=\frac{1}{N}\sum_{k=1}^{N}\left(\frac{d^{2}{\hat{z}}_{k}}{d\Omega_{k}^{2}}+\frac{0.5}{\Omega_{k}}\frac{d{\hat{z}}_{k}}{d\Omega_{k}}+e^{2{\hat{z}}_{k}}-0.5e^{{\hat{z}}_{k}}\right)+\frac{1}{2}\left({\left({\hat{z}}_{0}-\log(2)\right)}^{2}+{\left({\hat{z}}_{N}\right)}^{2}\right)$$

Optimization is performed through the hybridization of GA-SQPM to calculate the numerical representations of problem 1. The numerical outcomes are derived in Table 2, Table 3, Table 4 and Table 5 for small and large neurons three, 10, and 15, respectively, using a 0.05 step size with input [0,1]. One can observe that the proposed and exact solutions for three, 10, and 15 neurons consistently overlap the exact solutions. It is also noticed that, by taking a small number of neurons, the performance of ANN-GA-SQPM is reasonably good, but greater accuracy is observed for larger, 15 neuron-based networks. However, the complexity cost increases by increasing the number of variables/neurons in the networks.

**Problem 2:** Consider the following singular Lane–Emden nonlinear model along with the Dirichlet boundary conditions, which are written as:

(12) $$\begin{array}{l} \frac{d^{2}z}{d\Omega^{2}}+\frac{0.5}{\Omega }\frac{dz}{d\Omega }-\Omega^{2}e^{z}(16\Omega^{4}e^{z}-14)=0,\\ z(0)=\log(0.25),\;\;z(1)=\log(0.2). \end{array}$$

The exact solution for the above equation is $\ln\left(\frac{1}{4+\Omega^{4}}\right)$. The error function is given as:

(13) $$\begin{array}{l} \xi_{Fit}=\frac{1}{N}\sum_{k=1}^{N}\left(\frac{d^{2}{\hat{z}}_{k}}{d\Omega_{k}^{2}}+\frac{0.5}{\Omega_{k}}\frac{d{\hat{z}}_{k}}{d\Omega_{k}}-\Omega_{k}^{2}e^{{\hat{z}}_{k}}(16\Omega_{k}^{4}e^{{\hat{z}}_{k}}-14)\right)\\ \;\;\;\;\;\;\;\;+\frac{1}{2}\left({\left({\hat{z}}_{0}-\log(0.25)\right)}^{2}+{\left({\hat{z}}_{N}-\log(0.2)\right)}^{2}\right), \end{array}$$

**Problem 3:** Consider the following singular Lane–Emden nonlinear model along with Neumann boundary conditions is written as:

(14) $$\begin{array}{l} \frac{d^{2}z}{d\Omega^{2}}+\frac{2}{\Omega }\frac{dz}{d\Omega }-e^{z}(4\Omega^{2}e^{z}-6)=0,\\ z^{\prime }(0)=0,\;\;z^{\prime }(1)=-0.4. \end{array}$$

The exact solution for the above equation is $\ln\left(\frac{1}{4+\Omega^{2}}\right)$. The error function is given as:

(15) $$\xi_{Fit}=\frac{1}{N}\sum_{k=1}^{N}\left(\frac{d^{2}{\hat{z}}_{k}}{d\Omega_{k}^{2}}+\frac{2}{\Omega_{k}}\frac{d{\hat{z}}_{k}}{d\Omega_{k}}-e^{{\hat{z}}_{k}}(4\Omega_{k}^{2}e^{{\hat{z}}_{k}}-6)\right)\;\;+\frac{1}{2}\left({\left({{\hat{z}}^{\prime }}_{0}\right)}^{2}+{\left({{\hat{z}}^{\prime }}_{N}+0.4\right)}^{2}\right),$$

**Problem 4:** Consider the following singular Lane–Emden nonlinear model along with Neumann–Robin boundary conditions used in the modelling of isothermal gas spheres is given as:

(16) $$\begin{array}{l} \frac{d^{2}z}{d\Omega^{2}}+\frac{2}{\Omega }\frac{dz}{d\Omega }+z^{5}=0,\\ z^{\prime }(0)=0,\;\;z(1)=\sqrt{0.75}. \end{array}$$

The exact solution the for above equation is $\sqrt{\frac{3}{3+\Omega^{2}}}$. The error function is given as:

(17) $$\xi_{Fit}=\frac{1}{N}{\sum_{k=1}^{N}\left(\frac{d^{2}{\hat{z}}_{k}}{d\Omega_{k}^{2}}+\frac{2}{\Omega_{k}}\frac{d{\hat{z}}_{k}}{d\Omega_{k}}+z_{k}^{5}\right)}^{2}+\frac{1}{2}\left({\left({{\hat{z}}^{\prime }}_{0}\right)}^{2}+{\left({\hat{z}}_{N}-\sqrt{0.75}\right)}^{2}\right),$$

## 4. Investigation through Multiple Executions of ANN-GA-SQPM

The proposed results through ANN-GA-SQPM for fifty independent trials to accomplish the system parameter for the singular models that involve Neumann–Robin, Dirichlet, and Neumann boundary conditions are given in Equations in set (3). The best weights set is applied to designate the obtained results of the singular model, being mathematically given as:

(18) $$\begin{array}{l} {\hat{z}}_{1}(\Omega )=\frac{-3.7679}{1+e^{-(-3.012\Omega -3.285)}}+\frac{0.0376}{1+e^{-(-0.388\Omega -0.334)}}+\frac{1.1037}{1+e^{-(-0.173\Omega -3.2126)}}+\\ \;\;\;\;\;\;\;\;\;\;\;\;\;\;\;\;\;\ldots -\frac{0.6175}{1+e^{-(\;0.222\Omega +1.136)}}, \end{array}$$

(19) $$\begin{array}{l} {\hat{z}}_{2}(\Omega )=\frac{-2.1015}{1+e^{-(-4.269\Omega -13.333)}}-\frac{5.5141}{1+e^{-(4.496\Omega +11.462)}}+\frac{8.9057}{1+e^{-(-7.248\Omega -12.503)}}+\\ \;\;\;\;\;\;\;\;\;\;\;\;\;\;\;\;\;\;\ldots -\frac{6.4430}{1+e^{-(\;0.073\Omega +1.069)}}, \end{array}$$

(20) $$\begin{array}{l} {\hat{z}}_{3}(\Omega )=\frac{5.5275}{1+e^{-(17.177\Omega +19.987)}}-\frac{0.9686}{1+e^{-(0.297\Omega +14.586)}}-\frac{13.6035}{1+e^{-(1.585\Omega +18.439)}}+\\ \;\;\;\;\;\;\;\;\;\;\;\;\;\;\;\;\;\;\ldots -\frac{1.4923}{1+e^{-(\;1.740\Omega -11.650)}}, \end{array}$$

(21) $$\begin{array}{l} {\hat{z}}_{4}(\Omega )=\frac{1.4575}{1+e^{-(1.003\Omega -10.567)}}+\frac{0.2586}{1+e^{-(4.234\Omega +1.983)}}-\frac{3.32035}{1+e^{-(1.679\Omega -9.102)}}+\\ \;\;\;\;\;\;\;\;\;\;\;\;\;\;\;\;\;\;\ldots -\frac{3.943}{1+e^{-(\;10.219\Omega +10.745)}}, \end{array}$$

**Figure 1:**
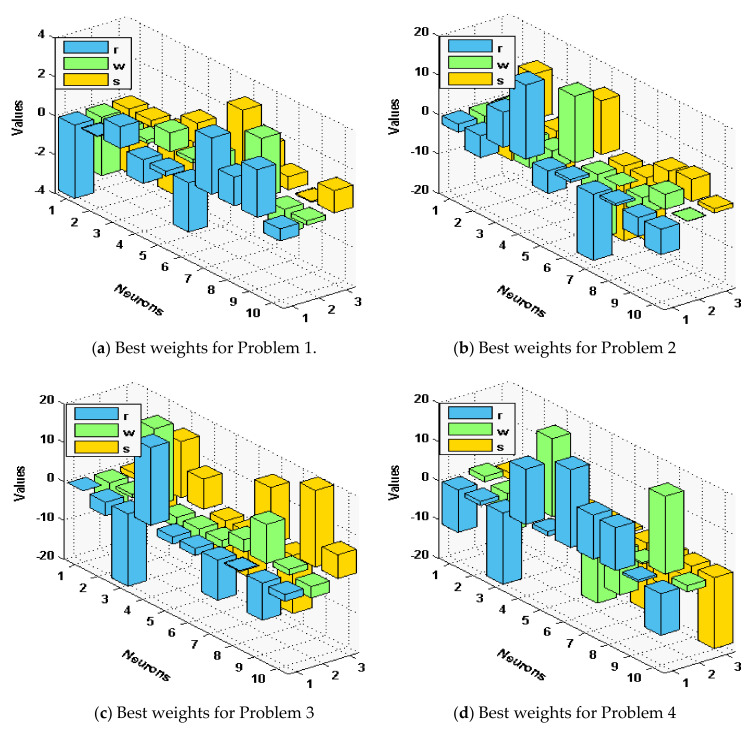
Best weights to solve the singular model of Lane–Emden type.

The optimal performance is provided to solve the singular model that involves Dirichlet and Neumann boundary conditions for fifty runs. Figure 1 plots a set of best weights using 30 variables.

**Figure 2:**
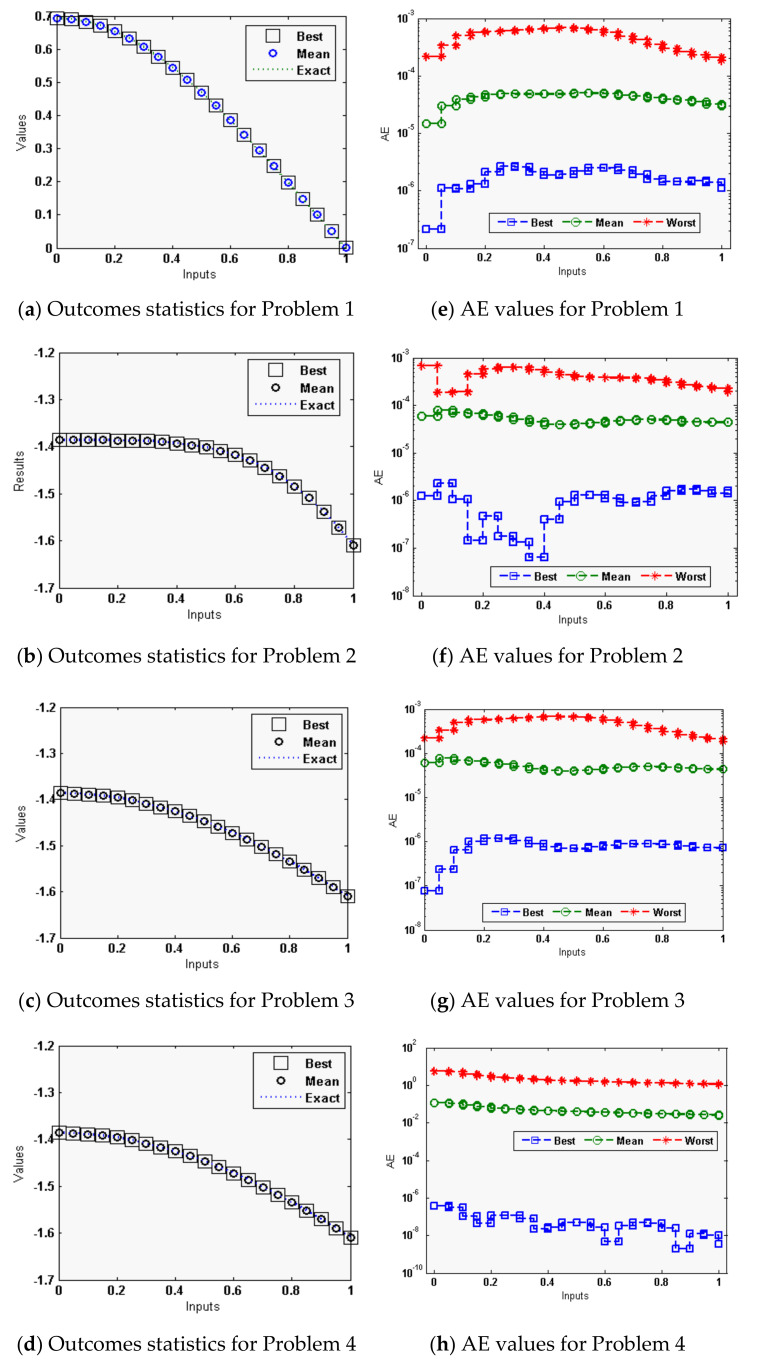
Comparison of results along with the AE through ANN-GA-SQPM to solve the singular model of Lane–Emden type. (**a**–**d**) for the solution dynamics, while (**e**–**h**) for AE.

**Figure 3:**
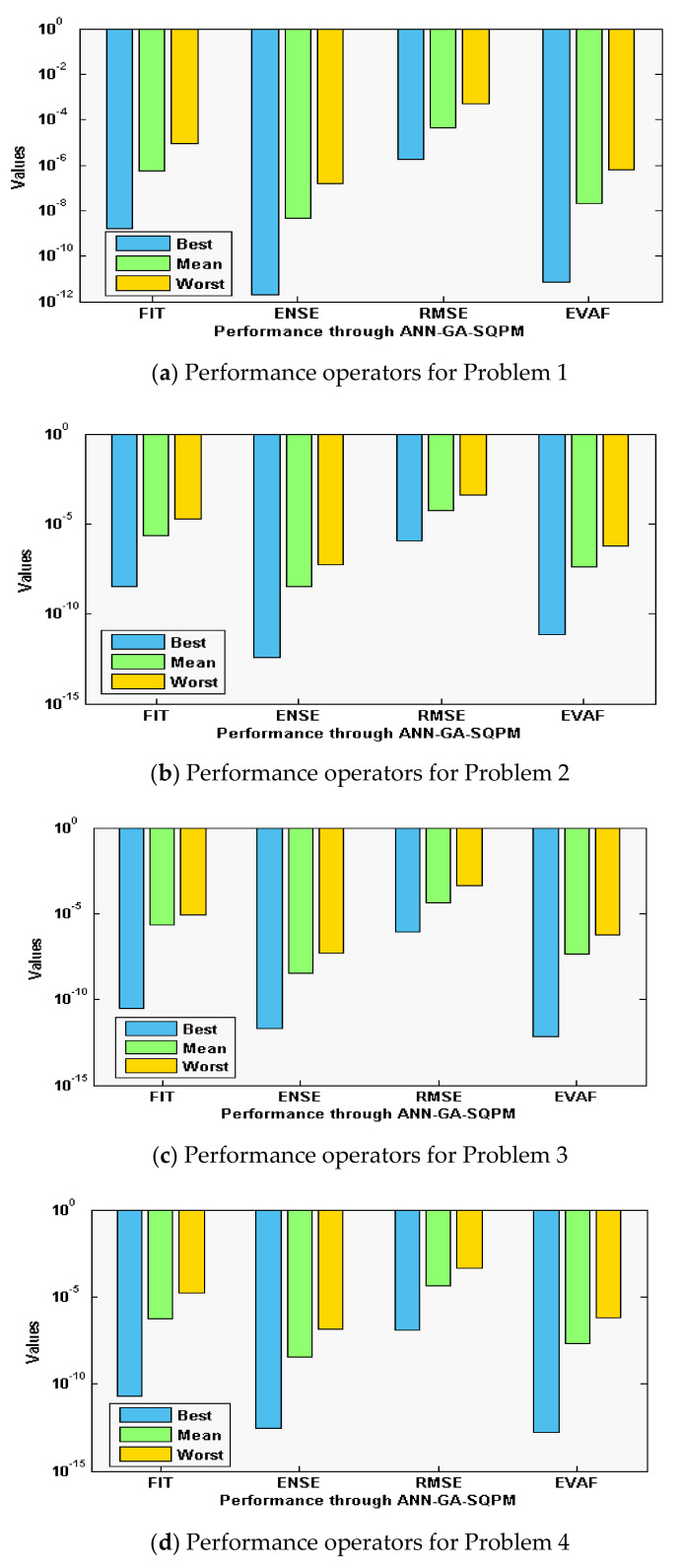
Performance measures through ANN-GA-SQPM to solve the singular model of Lane–Emden type.

The statistical measures of the ANN-GA-SQPM are accessible for solving the singular model of Lane Emden type, as defined in Equations in set (3), using 30 numbers of variables or 10 neurons, which is practicable in terms of complexity and accuracy as compared to three and 15 neurons. The ANN-GA-SQPM simulations have been accompanied by 50 trials to solve all four problems of those singular models, which involve Neumann–Robin, Dirichlet, and Neumann boundary conditions. The numerical results are provided on the basis of statistical performances that are graphically depicted in Figure 2 and Figure 3.

The exact, approximate results with minimum (Min) fitness (FIT) values, i.e., the best values, approximate outcomes with maximum (Max) FIT values, the worst, along with mean outcomes, are illustrated in Figure 2a–d for each singular models, which involve Neumann–Robin, Dirichlet, and Neumann boundary conditions. Nevertheless, the RMSE values at the same inputs are derived in Figure 2f–h. One can conclude that the proposed outcomes of the ANN-GA-SQPM attained a sensible precision even in the worst case too, although there is no perceptible difference in the presentation by deviation of the singular models that involve Neumann–Robin, Dirichlet and Neumann boundary conditions.

Moreover, the performance operatives for the FIT, RMSE, ENSE, and TIC are also considered, and their statistical based outcomes are provided in Figure 3a–d for each problem of the singular model. These consequences authenticate the comparable tendencies of performance based different measures. For problem 1, the FIT, ENSE, RMSE, and EVAF best values lie around 10^−08^ to 10^−09^, 10^−11^ to 10^−12^, 10^−05^ to 10^−06^, and 10^−10^ to 10^−11^. The mean values for problem 1 are found around 10^−06^ to 10^−07^, 10^−08^ to 10^−09^, 10^−04^ to 10^−05^, and 10^−07^ to 10^−08^. The worst values even lie around 10^−04^ to 10^−05^, 10^−06^ to 10^−07^, 10^−03^ to 10^−04^, and 10^−06^ to 10^−07^. For problem 2, the FIT, ENSE, RMSE, and EVAF best values lie around 10^−09^ to 10^−10^, 10^−12^ to 10^−14^, 10^−05^ to 10^−07^, and 10^−11^ to 10^−13^. The mean values for problem 2 are found around 10^−05^ to 10^−07^, 10^−09^ to 10^−10^, 10^−04^ to 10^−05^, and 10^−06^ to 10^−08^. The worst values even lie around 10^−04^ to 10^−05^, 10^−07^ to 10^−08^, 10^−03^ to 10^−05^, and 10^−06^ to 10^−07^. For problem 3, the FIT, ENSE, RMSE, and EVAF best values lie around 10^−10^ to 10^−11^, 10^−12^ to 10^−13^, 10^−06^ to 10^−08^, and 10^−12^ to 10^−14^. The mean values for problem 3 are found around 10^−05^ to 10^−06^, 10^−08^ to 10^−10^, 10^−04^ to 10^−05^, and 10^−06^ to 10^−07^. The worst values even lie around 10^−04^ to 10^−05^, 10^−06^ to 10^−08^, 10^−02^ to 10^−03^, and 10^−06^ to 10^−08^. For problem 4, the FIT, ENSE, RMSE, and EVAF best values lie around 10^−10^ to 10^−11^, 10^−13^ to 10^−14^, 10^−06^ to 10^−07^, and 10^−13^ to 10^−14^. The mean values for problem 4 are found around 10^−05^ to 10^−07^, 10^−08^ to 10^−09^, 10^−06^ to 10^−07^, and 10^−07^ to 10^−08^. The worst values even lie around 10^−04^ to 10^−05^, 10^−06^ to 10^−08^, 10^−04^ to 10^−05^, and 10^−05^ to 10^−07^. These optimal close values for each operator enhance the worth of the propose ANN-GA-SQPM for solving the nonlinear singular Lane–Emden system.

The complexity measures of ANN-GA-SQPM are shown in terms of time, iterations, and FIT assessed during the optimization procedures of hybrid heuristics GA-SQPM and standalone GAs to adjust the network’s decision variables. The optimization performance of GAs is degraded considerably with the increase of generations and rapid convergence achieved with the SQPM procedure, but at the cost of additional computations. The outcomes through the complexity indices are provided in Table 6 for each problem of the singular model of the Lane–Emden type for GA-SQPM. The hybrid heuristics GA-SQPM take almost 20% more computation time, i.e., 402, 805, 813, and 354 time consumed for ANN-GA for problems 1, 2, 3, and 4, respectively. Moreover, the computational time taken by three, 10, and 15 based neuron networks are found around 100 ± 10, 410 ± 300, and 750 ± 450. One may observe that no perceptible difference is perceived while using these complexity operatives with a fixed neuron for ANN-GA-SQPM to solve the singular model of Lane–Emden type that approves the smooth execution of the proposed approach for different problems.

## 5. Performance Operators

In this section, the performances that are based on RMSE, ENSE, and EVAF are provided. The mathematical measures of these operators are given as:

(22) $$\mathrm{RMSE}=\left[\sqrt{\frac{1}{n}\sum_{m=1}^{n}{\left(\Psi_{m}-{\hat{\Psi }}_{m}\right)}^{2}}\right].$$

(23) $$\left\{\begin{array}{l} VAF=\left(1-\frac{\mathrm{var}\left(Z_{i}(T)-{\hat{Z}}_{i}(T)\right)}{\mathrm{var}\left(Z_{i}(T)\right)}\right)\times 100,\\ EVAF=\left|VAF-100\right|, \end{array}\right.$$

(24) $$\mathrm{NSE}=\left\{1-\frac{{\sum_{m=1}^{n}\left(\Psi_{m}-{\hat{\Psi }}_{m}\right)}^{2}}{{\sum_{m=1}^{n}\left(\Psi_{m}-{\bar{\Psi }}_{m}\right)}^{2}},\right.\;\;\;{\bar{\Psi }}_{m}=\frac{1}{n}\sum_{m=1}^{n}\Psi_{m},$$

(25) ENSE=1−NSE

## 6. Conclusions

The present study is aims to design an alternate, stable, and accurate stochastic computing numerical approach to solve the singular model of Lane–Emden type that involves Neumann–Robin, Dirichlet, and Neumann boundary conditions by manipulating the ANN, local search SQPM, and GA based global search. The proposed structure of ANN-GA-SQPM is examined for different neurons/variables in the system and the presentation is acquired reasonably for all neurons based on the networks of ANN. The most reliable and accurate solutions are attained for large neurons, but the complexity increased. Statistics results through different performances for the convergence, precision, and complexity authenticate the value of the proposed ANN-GA-SQPM to solve the singular model of the Lane–Emden type of problem that involves Neumann–Robin, Dirichlet, and Neumann boundary conditions.

In the future, one may solve the fractional form of the singular models with Neumann–Robin, Dirichlet, and Neumann boundary conditions using the proposed ANN-GA-SQPM. Additionally, the memetic computing paradigm of ANN-GA-SQPM can be a good alternative to be exploited for problems involving the study of sensors [47,48,49,50,51].

## Figures and Tables

**Table 1:** Pseudocode of the process of optimization using the ANN-GA-SQPM.

**Start of GA**
Inputs:
The chromosome with the same entries of the system are signified as:
W=[r,w,s]
Population: The chromosomes set is designated as:
$r=[r_{1},r_{2},r_{3},\ldots r_{k}],\;\;w=[w_{1},w_{2},w_{3},\ldots ,w_{k}]$ and $s=[s_{1},s_{2},s_{3},\ldots ,s_{k}]$
$P={\left[W_{1},\;\;W_{2},,\ldots ,W_{k}\right]}^{t}$
Output: The best weights of GA are *W*_Best-GA_
Initialization
Create *W* that is a *W*_Best-GA_ of real numbers to signify a chromosome. Initialize the *W* with real entries. Adjust the ‘Generation’ & ‘declarations’ values of ‘gaoptimset’ & GA routines
Fitness formulation
Accomplish the $\xi_{Fit}$ in *P* to show all *W* for Equations (5)–(8)
Termination
Stop the process to accomplish
• $\xi_{Fit}$= 10 ^−18^, TolFun = 10^−21^, Generations = 100, , • TolCon = 10^−22^, Population Size = 270, StallGenLimit = 120
Go to [storage], when stopping standards obtains.
Ranking
Rank *W* of *P* for brilliance of $\xi_{Fit}$
Storage
Save *W*_Best-GA_, iterations, $\xi_{Fit}$ and time for the current trials of GAs
End of GA
GA-SQPM Start
Inputs
*W*_Best-GA_ is the start point
Output
*W*_GA-SQPM_ represents the best values
Initialize
Adjust *W*_GA-SQPM_ represents an initial input
Termination
Stop the procedure, when $\xi_{Fit}$= 10^−^^18′^, generations = 1000, TolFun = 10^−21^,
TolX=10^−^^19′^, TolCon =10^−18′^, MaxEvalsFun= 229,000
While [Terminate]
Fitness Calculations
Calculate $\xi_{Fit}$ of the present *W* using Equations (5–8).
Amendments
Invoke ‘fmincon’ for the SQPM. Adjust *W* for each generation of SQPM. Calculate
Calculate $\xi_{Fit}$ of updated *W* using Equations (5–8)
Accumulate
Store *W*_GA-SQPM_, time, $\xi_{Fit}$ and number of generations for the current trials of SQPM.
End of GA-SQPM Procedure

**Table 2:** Results comparison of Problem 1 based on ANN-GA-SQPM for three, 10, and 15 neurons, or nine, 30, and 45 variables with reference solutions.

Ω	Exact	Approximate Results $\hat{z}(\Omega )$		
	$\hat{z}(\Omega )$	9 Variables	30 Variables	45 Variables
0	0.69314718056	0.68224453346	0.69315090195	0.69314891025
0.05	0.69065030036	0.67524281241	0.69064571170	0.69064852047
0.1	0.68319684971	0.66423337183	0.68320463422	0.68319977498
0.15	0.67089657163	0.64935335193	0.67091717339	0.67090442363
0.2	0.65392646741	0.63075628397	0.65395396612	0.65393705056
0.25	0.63252255874	0.60861130029	0.63255161225	0.63253391938
0.3	0.60696948432	0.58310217609	0.60699764007	0.60698071676
0.35	0.57758883993	0.55442621042	0.57761623704	0.57759992784
0.4	0.54472717544	0.52279295679	0.54475520147	0.54473850462
0.45	0.50874445756	0.48842281690	0.50877439795	0.50875637605
0.5	0.47000362925	0.45154551423	0.47003584924	0.47001621283
0.55	0.42886168591	0.41239846634	0.42889548155	0.42887471647
0.6	0.38566248081	0.37122507743	0.38569646043	0.38567556481
0.65	0.34073129354	0.32827297406	0.34076400945	0.34074402602
0.7	0.29437106060	0.28379220851	0.29440158495	0.29438315629
0.75	0.24686007793	0.23803345472	0.24688828252	0.24687142635
0.8	0.19845093872	0.19124622183	0.19847736564	0.19846157782
0.85	0.14937045665	0.14367710991	0.14939582945	0.14938048998
0.9	0.09982033528	0.09556813137	0.09984493662	0.09982983598
0.95	0.04997836981	0.04715511977	0.05000168636	0.04998732054
1	0	0.00133375423	0.00002119692	0.00000831368

**Table 3:** Results comparison of Problem 2 using ANN-GA-SQPM based on three, 10, and 15 neurons or nine, 30, and 45 variables neurons with the reference solutions.

Ω	Exact	Approximate Results $\hat{z}(\Omega )$		
	$\hat{z}(\Omega )$	9 Variables	30 Variables	45 Variables
0	−1.38629436112	−1.38629436112	−1.38619926024	−1.38630126484
0.05	−1.38629592362	−1.38629592362	−1.38611885693	−1.38631522992
0.1	−1.38631936081	−1.38631936081	−1.38617352354	−1.38633499755
0.15	−1.38642091561	−1.38642091561	−1.38633380823	−1.38642949431
0.2	−1.38669428114	−1.38669428114	−1.38665342802	−1.38669799402
0.25	−1.38727044709	−1.38727044709	−1.38725068090	−1.38727265740
0.3	−1.38831731357	−1.38831731357	−1.38829571544	−1.38832031881
0.35	−1.39003890406	−1.39003890406	−1.39000152175	−1.39004329283
0.4	−1.39267396808	−1.39267396808	−1.39261717627	−1.39267897863
0.45	−1.39649373274	−1.39649373274	−1.39642230631	−1.39649806188
0.5	−1.40179854766	−1.40179854766	−1.40172202250	−1.40180115494
0.55	−1.40891317854	−1.40891317854	−1.40884175300	−1.40891377590
0.6	−1.41818054998	−1.41818054998	−1.41812154180	−1.41817964531
0.65	−1.42995382621	−1.42995382621	−1.42990946826	−1.42995237211
0.7	−1.44458685387	−1.44458685387	−1.44455392788	−1.44458570299
0.75	−1.46242316947	−1.46242316947	−1.46239459698	−1.46242261605
0.8	−1.48378398240	−1.48378398240	−1.48375199787	−1.48378364332
0.85	−1.50895575599	−1.50895575599	−1.50891569341	−1.50895489962
0.9	−1.53817818786	−1.53817818786	−1.53813127387	−1.53817637114
0.95	−1.57163349586	−1.57163349586	−1.57158645455	−1.57163106694
1	−1.60943791243	−1.60943791243	−1.60939677357	−1.60943565431

**Table 4:** Results comparison of Problem 3 using ANN-GA-SQPM based on three, 10, and 15 neurons or nine, 30, and 45 variables with the reference solutions.

Ω	Exact	Approximate Results $\hat{z}(\Omega )$		
	$\hat{z}(\Omega )$	9 Variables	30 Variables	45 Variables
0	−1.38629436112	−1.48446054700	−1.38630356972	−1.38631710010
0.05	−1.38691916589	−1.48757921354	−1.38692665406	−1.38694119063
0.1	−1.38879124132	−1.49113183052	−1.38879709413	−1.38881050847
0.15	−1.39190359988	−1.49517102282	−1.39190920225	−1.39191976243
0.2	−1.39624469197	−1.49975342347	−1.39625061225	−1.39625835324
0.25	−1.40179854766	−1.50493923270	−1.40180467700	−1.40181070914
0.3	−1.40854497005	−1.51079149158	−1.40855102806	−1.40855665180
0.35	−1.41645977545	−1.51737500782	−1.41646560212	−1.41647178419
0.4	−1.42551507427	−1.52475487352	−1.42552068561	−1.42552789275
0.45	−1.43567958630	−1.53299452399	−1.43568510504	−1.43569335640
0.5	−1.44691898294	−1.54215330699	−1.44692454151	−1.44693355582
0.55	−1.45919624910	−1.55228356454	−1.45920191864	−1.45921127714
0.6	−1.47247205736	−1.56342727723	−1.47247782108	−1.47248710477
0.65	−1.48670514705	−1.57561238316	−1.48671091461	−1.48671979939
0.7	−1.50185270175	−1.58884895695	−1.50185835049	−1.50186665735
0.75	−1.51787071891	−1.60312551155	−1.51787614407	−1.51788384920
0.8	−1.53471436624	−1.61840575515	−1.53471952326	−1.53472673514
0.85	−1.55233832039	−1.63462618173	−1.55234324496	−1.55235015636
0.9	−1.57069708412	−1.65169487952	−1.57070187969	−1.57070870155
0.95	−1.58974527909	−1.66949189317	−1.58975006558	−1.58975694855
1	−1.60943791243	−1.68787136420	−1.60944273339	−1.60944968143

**Table 5:** Results comparison of Problem 4 using ANN-GA-SQPM based on three, 10 and 15 neurons or nine, 30, and 45 variables with the reference solutions.

Ω	Exact	Approximate Results $\hat{z}(\Omega )$		
	$\hat{z}(\Omega )$	9 Variables	30 Variables	45 Variables
0	1.00000000000	0.99488248094	1.00000783826	1.00000105336
0.05	0.99958359357	0.99454671429	0.99959083004	0.99958445423
0.1	0.99833748846	0.99326955610	0.99834175320	0.99833784278
0.15	0.99627096277	0.99110494864	0.99627165084	0.99627085144
0.2	0.99339926780	0.98810559022	0.99339689664	0.99339889181
0.25	0.98974331861	0.98432281012	0.98973898250	0.98974290152
0.3	0.98532927816	0.97980646561	0.98532421813	0.98532898386
0.35	0.98018805078	0.97460485937	0.98018334848	0.98018795036
0.4	0.97435470369	0.96876467525	0.97435109794	0.97435478017
0.45	0.96786783699	0.96233093074	0.96786565238	0.96786801419
0.5	0.96076892283	0.95534694435	0.96076809221	0.96076910439
0.55	0.95310163425	0.94785431626	0.95310179112	0.95310174015
0.6	0.94491118252	0.93989292085	0.94491179604	0.94491117389
0.65	0.93624367977	0.93150090948	0.93624420395	0.93624356674
0.7	0.92714554082	0.92271472236	0.92714555080	0.92714537307
0.75	0.91766293548	0.91356910830	0.91766222690	0.91766277924
0.8	0.90784129900	0.90409715099	0.90783993125	0.90784120821
0.85	0.89772490592	0.89433030113	0.89772317564	0.89772489740
0.9	0.88735650942	0.88429841320	0.88735484682	0.88735655314
0.95	0.87677704604	0.87402978614	0.87677583278	0.87677708125
1	0.86602540378	0.86355120724	0.86602471655	0.86602539000

**Table 6:** The complexity performance through ANN-GA-SQPM for each example of the singular model of Lane–Emden type.

Problem	Implementation Time		Iterations		Count of Function	
	Min	SD	Min	SD	Min	SD
1	522.78467	2576.40890	433.30000	110.29834	27589.96000	7079.39701
2	861.83918	5412.52809	472.52000	73.426410	29664.62000	4479.75041
3	881.232020	26.2453000	467.16000	104.07147	30003.56000	6967.51609
4	437.11324	2576.23782	338.94000	147.12974	22032.32000	9767.47337

## Data Availability

Not applicable.
